# Assessment of Optimal Screening Tests for the Detection of an Inflammatory Myositis-associated Interstitial Lung Disease

**DOI:** 10.7759/cureus.7875

**Published:** 2020-04-28

**Authors:** Sonia Castillo, Brietta Forbes, John Chen, Mark J Hamblin

**Affiliations:** 1 Pulmonary and Critical Care Medicine, Kansas City Veteran Affairs Medical Center, Kansas City, USA; 2 Internal Medicine / Pulmonary and Critical Care, Menorah Hospital, Overland Park, USA; 3 Internal Medicine, Kansas University Medical Center, Kansas City, USA; 4 Internal Medicine / Pulmonary and Critical Care, University of Kansas Medical Center, Kansas City, USA

**Keywords:** interstitial lung disease, myositis, aldolase, creatine kinase, anti-ssa

## Abstract

Background

Interstitial lung disease (ILD) is a common pathologic consequence of the idiopathic inflammatory myopathies, and it may be the initial presentation of autoimmune disease in many cases. There are no well-established guidelines to direct the evaluation of this disease in these cases. This study looked at the utility of four common serologic tests to screen for a myositis-associated ILD.

Methods

This is a single institution retrospective analysis of four common serologic tests (antinuclear antibody [ANA], creatine kinase [CK], aldolase, and anti-Sjögren's syndrome A [anti-SSA]) to detect a positive antibody on an extended myositis antibody panel.

Results

The serum aldolase was the most sensitive test to detect the presence of a positive antibody on an extended myositis antibody panel with a sensitivity of 54.5%. The anti-SSA was the least sensitive at 21.4%. A positive result for anti-SSA antibodies was associated with a 100% positive predictive value when all other screening tests (ANA, aldolase, and CK) were also positive.

Conclusion

No single screening test was sufficient for the evaluation of a myositis-associated ILD. A positive serum aldolase had higher sensitivity, and a positive SSA had a high positive predictive value when other screening markers were also elevated, but clinicians still need to maintain a high index of suspicion for myositis-associated ILD.

## Introduction

The idiopathic inflammatory myopathies (IIM) are a group of chronic, autoimmune disorders affecting predominantly proximal muscle groups, including necrotizing autoimmune myopathy, inclusion body myopathies, polymyositis (PM), dermatomyositis (DM) and the sub-classification of anti-synthetase syndromes (ASS). Lung involvement, particularly interstitial lung disease (ILD), is a common complication of these autoimmune disorders, especially in the presence of an anti-synthetase antibody [1]. In most cases, when ILD occurs, it can be the initial manifestation of the disease, or it occurs simultaneously with mild or even subclinical myopathy [2, 3]. More common clinical features of hyperkeratosis, Raynaud’s phenomenon, or classic signs of DM may be subtle and overlooked without experience in diagnosing these syndromes. Patients with more rapid presentations may be admitted directly to hospitals and intensive care units, bypassing specialty clinics with more experience in diagnosing and managing these diseases. In cases of more rapidly progressive ILD, such as clinically amyopathic myositis, an antinuclear antibody (ANA) may be low or even negative and clinical features of myopathy may be absent. In this setting, if a more extensive autoimmune evaluation hinges on a positive ANA, the appropriate diagnosis may be missed entirely leading to inadequate treatment or potentially unnecessary procedures, such as a lung biopsy, which may invoke additional risks. 

Currently, there is no consensus to guide clinicians in the evaluation of these lung dominant presentations. The Bohan and Peter criteria used to guide the diagnosis of PM and DM may have limited value if clinical features are mild or absent [4]. Muscle enzyme testing can be useful, but acute presentations of lung dominant disease often do not show elevations in the creatine kinase (CK), but aldolase may be elevated due to immune-mediated injury predominantly affecting the early regenerative myocytes [5]. Even if a positive ANA leads to a more extensive work-up for connective tissue disease (CTD), clinicians may not send an extended myositis antibody panel without other clinical or laboratory features to support this decision. 

The purpose of this study was to assess the positive predictive value of various tests in the setting of a lung dominant inflammatory myopathy, to detect a specific myositis-associated antibody (MAA) or an anti-synthetase antibody on an extended myositis antibody panel.

## Materials and methods

**Figure 1:**
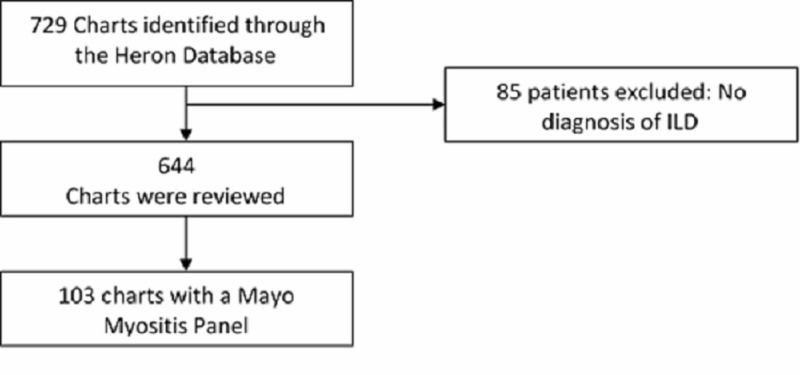
Flowchart of patients who had a Mayo Myositis Panel ILD - interstitial lung disease

We performed a retrospective review of patient records seen at the University of Kansas Hospital for ILD evaluation between July 2012 and December 2017. This study was approved by the University of Kansas Institutional Review Board (IRB) with the number 141251. The need for written consent was waived by the IRB due to the retrospective nature of the study. We further identified patients who had an extended myositis antibody panel sent for evaluation of their ILD. During this five-year period, there were 644 patients identified with ILD, and 103 patients had an extended myositis antibody panel (also known as Mayo Myositis Panel) ordered, as part of the workup for a new diagnosis of ILD (Figure 1). At least one positive MAA was detected in 44 patients. We examined commonly ordered tests including ANA, CK, aldolase and anti-SSA (anti-Sjögren's syndrome A), assessing the sensitivity, specificity, positive and negative predictive values. Statistical analysis was performed using SAS version 9.4 (SAS Institute, Cary, US).

## Results

Demographics

The mean age at the time of diagnosis of a myositis-associated ILD was 61, with a relatively even distribution between male and female patients (50.5% and 49.5%, respectively; Table 1). 

**Table 1:** Demographics MAA - myositis-associated antibody

	All patients n=103	Patients with at least one positive MAA n=44	Patients with negative MAA n=59	p
Age (years)	61.2 ± 13.5	58.9 ± 14.7	62.9 ± 12.5	0.14
Male gender (%)	50.5	50.0	50.1	0.093
Alive (%)	72.8	77.3	69.5	0.38

At our institution, there is a screening ILD panel which includes erythrocyte sedimentation rate (ESR), C-reactive protein (CRP), ANA, rheumatoid factor (RF), anti-cyclic citrullinated peptide (anti-CCP), perinuclear antineutrophil cytoplasmic antibody (P-ANCA), cytoplasmic antineutrophil cytoplasmic antibodies (C-ANCA), hypersensitivity pneumonitis panel, CK, aldolase, the anti-histidyl-tRNA synthetase antibody (anti-Jo1), and anti-Sjögren's syndrome A/anti-Sjögren's syndrome B (anti-SSA/SSB). A second panel for a more comprehensive autoimmune evaluation includes serum anti-topoisomerase I (anti-SCL-70), anti-double stranded DNA (anti-dsDNA), anti-ribonucleoprotein/Smith (anti-RNP/Sm), myeloperoxidase-antineutrophil cytoplasmic antibody (MPO-ANCA), proteinase 3 anti-neutrophil cytoplasmic antibody (PR3-ANCA), anti-centromere as well as an extended myositis antibody panel that includes: anti-Sjögren's syndrome A/Ro protein 52 (anti-SSA/Ro52), anti-Jo1, anti-threonyl-tRNA synthetase (anti-PL7), anti-glycyl tRNA synthetase (anti-EJ), anti-isoleucyl-tRNA synthetase (anti-OJ), anti-Ku, anti-Mi-2, anti-signal recognition particle (anti-SRP), anti-exosome or anti-polymyositis scleroderma (anti-PM/Scl), anti-transcription intermediary factor gamma (anti-TIF1-GAMMA), anti-melanoma differentiation-associated gene 5 (anti-MDA5), anti-nuclear matrix protein 2 (anti-NXP2), anti-U1 ribonucleoprotein (anti-U1RNP), anti-U2 ribonucleoprotein (anti-U2RNP), and anti-U3 ribonucleoprotein (anti-U3RNP).

Following an initial chart review (excluding an ESR or CRP), the most commonly identified laboratory tests on our screening panel associated with a positive MAA were ANA, CK, aldolase, and anti-SSA.

**Figure 2:**
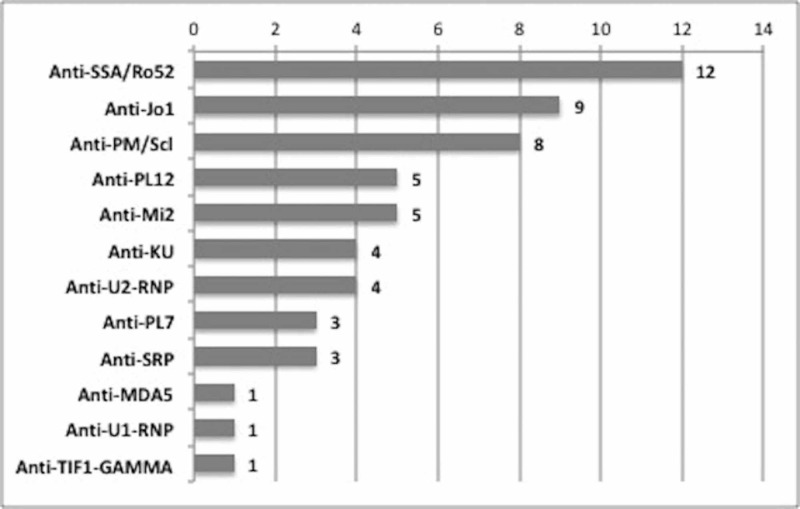
Antibodies identified on the extended myositis antibody panel (numbers represent number of patients) Anti-SSA/Ro52 - anti-Sjögren's syndrome A/Ro protein 52; Anti-Jo1 - the anti-histidyl-tRNA synthetase antibody; Anti-PM/Scl - anti-exosome or anti-polymyositis scleroderma; Anti-U2-RNP - anti-U2 ribonucleoprotein; Anti-PL7 - anti-threonyl-tRNA synthetase; Anti-SRP - anti-signal recognition particle; Anti-MDA5 - anti-melanoma differentiation-associated gene 5; Anti-U1-RNP - anti-U1 ribonucleoprotein; Anti-TIF1-GAMMA - anti-transcription intermediary factor gamma

The most commonly noted MAA on the 103 patients who had an extended myositis antibody panel were anti-SSA/Ro52 antibody (11.7%), anti-Jo1 (8.7%), and anti-PM/Scl (7.8%) as demonstrated on Figure 2.

ANA analysis

ANA was ordered in 100 patients. A positive ANA was noted in 52 subjects (52.0%). From these 52 patients, 26 had a positive MAA (26.0%). Of those patients with a positive ANA, five had an ANA titer of 1:320 or less. There were 17 patients with a negative ANA who ultimately had a positive MAA on the extended myositis antibody panel (17.0%). These results indicated a sensitivity of 60.5%, a specificity of 54.4%, a positive predictive value (PPV) of 50.0% and a negative predictive value (NPV) of 64.6%.

CK analysis

CK was ordered in 100 patients. A positive CK was noted in 28 patients (28.0%); 16 of these 28 patients (16.0%) had a positive MAA on the extended myositis antibody panel. In 12 patients (12.0%), a positive CK did not lead to the detection of a positive antibody on the extended myositis antibody panel. From those with a negative CK (72 patients), 26 had a positive MAA (26.0%). The CK results indicated a sensitivity of 38.1%, a specificity of 79.3%, a PPV of 57.1% and a negative predictive value NPV of 63.9%.

Aldolase analysis

Aldolase was ordered in all 103 patients. A positive aldolase was noted in 57 patients (55.3%). Of those, 24 patients (23.3%) had a positive MAA. In 33 patients (32.0%), a positive aldolase did not lead to the detection of a positive antibody on the extended myositis antibody panel. There were 20 patients with a negative aldolase who ultimately had a positive MAA (19.4%). The aldolase results indicated a sensitivity of 54.5%, a specificity of 44.1%, a PPV of 42.1%, and an NPV of 56.5%. 

Anti-SSA analysis

An anti-SSA was ordered in 101 patients. A positive anti-SSA was noted in 10 patients (10.0%). Of those, nine patients (9.0%) had a more specific antibody identified on the extended myositis antibody panel. In one patient (1.0%), a positive anti-SSA did not lead to the detection of a positive MAA. There were 33 patients with a negative anti-SSA who ultimately had a positive antibody on the extended myositis antibody panel (32.0%). The anti-SSA results indicated a sensitivity of 21.4%, a specificity of 98.3%, a PPV of 90.0%, and NPV of 63.7%. 

Combination analysis

We further analyzed various combinations of the ANA, CK, aldolase, and anti-SSA to determine an optimal screening profile to yield the highest sensitivity, specificity, positive, and negative predictive values.

**Aldolase + CK:** if CK and aldolase were both positive, the sensitivity was 31.8%, with a specificity of 84.7%. This correlated to a PPV of 60.9% and NPV of 62.5%.

**Aldolase + anti-SSA:** if aldolase and anti-SSA were both positive, the sensitivity was 13.6%, with a specificity of 98.3%. This correlated to a PPV of 85.7% and NPV of 60.4%.

**CK + anti-SSA:** if both CK and anti-SSA were positive, the sensitivity was 15.9% with a specificity of 100%. This correlated to a PPV of 100% and NPV of 61.5%.

**Aldolase + CK + anti-SSA:** if all these three tests were positive, the sensitivity was 13.6%, with a specificity of 100%. This correlated to a PPV of 100% and NPV of 60.8%.

**Aldolase + CK + ANA:** if both muscle enzymes and ANA were positive, the sensitivity was 22.7%, with a specificity of 96.6%. This correlated to a PPV of 83.3% and NPV of 62.6%.

**Aldolase + CK + ANA + anti-SSA:** if all four screening tests were positive, the sensitivity decreased to 9.1%. The specificity was 100% with a 100% PPV, and a 59.6% NPV.

**Any positive lab:** if any of the four screening tests were positive, the sensitivity increased to 86.4%. Specificity declined to 16.9%. The PPV was 43.7%, and the NPV was 62.5%.

## Discussion

The diagnosis of myositis-associated ILD (MA-ILD) can be challenging on many levels. Lung dominant presentations often lack the classical signs and symptoms of PM or DM [5]. Even when these features are present, they may be subtle and easily overlooked by inexperienced practitioners. This may include very slight hyperkeratosis on the lateral edges of the fingers appearing no different than callouses, or atypical presentations, such as Raynaud’s phenomenon isolated to one or two digits, mimicking arterial stenosis rather than classic Raynaud’s [6]. Pulmonary specialists are not always trained to detect these subtle clinical findings, but they are trained to evaluate ILD for a possible autoimmune etiology. Laboratory testing for serologic abnormalities often helps point the way to a connective tissue disease-associated interstitial lung disease (CTD-ILD). 

The existing guidelines for the work-up of an idiopathic interstitial pneumonitis recommend checking ANA, RF, anti-CCP and ANCA, with additional serologic testing as clinically indicated [7]. There is no guidance as to what other serologic tests to order and when, so there may be significant variation in the extent of the evaluation. In many cases, pulmonologists rely on rheumatologists to aid in the evaluation. Lung dominant myositis cases do not generally fit the Bohan and Peter criteria for the diagnosis of an inflammatory myopathy complicating rheumatologic evaluation. Frequently, if the ANA is low or negative, and if CK is normal, the evaluation may conclude that there is no autoimmune etiology for the ILD. This may lead to a lung biopsy, which carries the potential for 16% mortality if the ILD presentation is more rapidly progressive [8]. Sometimes empiric steroids for acute interstitial pneumonitis can make a difference, but there is also data to suggest that steroid therapy alone may be inadequate in these diseases. Thus, there is a clear need to identify best practices to help guide when to send an extended myositis antibody panel.

In this study, we perform a retrospective analysis of four of the most common screening tests used to aid in the detection of MA-ILD. In this analysis, we found inadequate sensitivity and specificity of these screening tests in isolation to detect a more specific MAA on an extended myositis panel. 

ANA was negative 37% of the time an MAA was present, and that percentage would increase to near 50% if low titer ANAs were disregarded. CK values performed more poorly, with 62.0% of patients having an MAA when the CK was within normal values. The specificity was superior at 79.3%, but that is not the goal of a screening test. Anti-SSA was the least sensitive at 21.4%, but it carried a very high specificity being the only screening test with a positive predictive value of over 90%, likely due to the higher prevalence of the anti-SSA/Ro52 antibody. Aldolase, which is often underutilized in these evaluations, carried a sensitivity of 54.5%. Unfortunately, this level of sensitivity is obviously too low to be used in isolation.

Consequently, we examined various combinations of these screening tests. Ultimately, if any of the four screening tests was positive, then the sensitivity rose to 86.4%. None of the laboratory tests in isolation, nor any combination, had a high enough negative predictive value to exclude a MA-ILD if results were negative. This indicates the need for a high index of clinical suspicion for MA-ILD, particularly when assessing a more rapidly progressive ILD, with a low threshold to send an extended myositis antibody panel. 

At some institutions, such as our own, pathology departments require justification for sending high-cost laboratory tests on hospitalized patients. The extended myositis antibody panel is a high-cost test (~$1300), only performed at a few centers in the United States. The combined costs of these four screening tests may be institution dependent, but they are relatively cheap in comparison to the commercial myositis panels, with the cost of the combination of laboratory tests estimated at ~$170 to $350 (aldolase $34-79; CK $23-59; ANA $33-68; and SSA $80-145 [9]). If the 100% positive predictive value noted when all four screening tests were positive is confirmed in other studies, it may negate the need to send a confirmatory myositis antibody panel, and it may provide clinicians a greater degree of confidence to move forward with treatment for MA-ILD in the right clinical context. This might also serve to prevent potentially risky or unnecessary lung biopsies in patients with more rapidly progressive ILD.

There are several limitations to our findings. This is a single institution study and depending on the assays utilized for the detection of laboratory tests, results could vary at other institutions. It has been reported that some commercial assays of the anti-SSA antibody may contain less than adequate amounts of Ro52 antibody, favoring Ro60 detection. We have seen this occur at our own institution, when a Ro52 antibody was noted to be moderate or high positive on an extended myositis antibody panel, despite a negative anti-SSA on our screening panel. We do not know if there is the potential for the Ro52 detection antibody to decay at a faster than expected rate over time in some commercially available enzyme-linked immunosorbent assays (ELISAs), or if there is another explanation for this finding. We also know that liver injury can be a contributory source of aldolase, and statins could cause elevations of both aldolase and CK. The retrospective nature of this study did not control for that possibility. If these patients were excluded that might alter results as well. Additionally, in some patients, ANA was obtained at an outside institution and may represent a direct rather than indirect ANA, which can be more sensitive for detection. Additionally, Sjögren’s patients were not evaluated in this cohort, but if they were included, we would see shifts in the specificity and PPV of the SSA results as well. Consequently, these findings need to be validated in a larger multi-institutional study in a controlled fashion. 

## Conclusions

In this single-institution retrospective analysis of patients diagnosed with MA-ILD, we demonstrated that screening for MA-ILD can be challenging. Clinicians should not rely on a single serologic test result to determine the probability of MA-ILD. Although the serum aldolase has a poor specificity, the increased sensitivity compared to CK values, suggests it should be considered as an important serologic marker utilized for screening of MA-ILD. In the absence of liver disease or statin use, it should prompt clinicians to send an extended myositis antibody panel. A positive anti-SSA antibody carries a high positive predictive value of disease, and a positive result should prompt empiric treatment in the appropriate clinical context, particularly when associated to other elevated screening tests such as ANA, CK and aldolase. Findings will need to be validated in a larger multi-center controlled study. This study provides strategies to improve detection efforts at presentation, but also indicates the need for clinicians to maintain a high index of suspicion for MA-ILD in the appropriate clinical context.

## References

[1] Katzap E, Barilla-LaBarca ML, Marder G (2011). Antisynthetase syndrome. Curr Rheumatol Rep.

[2] Labirua A, Lundberg IE (2010). Interstitial lung disease and idiopathic inflammatory myopathies: progress and pitfalls. Curr Opin Rheumatol.

[3] Koreeda Y, Higashimoto I, Yamamoto M (2010). Clinical and pathological findings of interstitial lung disease patients with anti-aminoacyl-tRNA synthetase autoantibodies. Intern Med.

[4] Leclair V, Lundberg IE (2018). New myositis classification criteria-what we have learned since Bohan and Peter. Curr Rheumatol Rep.

[5] Casciola-Rosen L, Hall JC, Mammen AL, Christopher-Stine L, Rosen A (2012). Isolated elevation of aldolase in the serum of myositis patients: a potential biomarker of damaged early regenerating muscle cells. Clin Exp Rheumatol.

[6] Chatterjee S, Prayson R, Farver C (2013). Antisynthetase syndrome: not just an inflammatory myopathy. Cleve Clin J Med.

[7] Travis WD, Costabel U, Hansell DM (2013). An official American Thoracic Society/European Respiratory Society statement: Update of the international multidisciplinary classification of the idiopathic interstitial pneumonias. American journal of respiratory and critical care medicine.

[8] Hutchinson JP, Fogarty AW, McKeever TM, Hubbard RB (2016). In-Hospital Mortality after Surgical Lung Biopsy for Interstitial Lung Disease in the United States. 2000 to 2011. Am J Respir Crit Care Med.

[9] (2020). Find lab tests online. https://www.findlabtest.com/lab-test/.

